# Antimicrobial resistance analysis of *Klebsiella pneumoniae* bloodstream infections based on a random forest algorithm: a longitudinal study based on data from tertiary hospitals in China from 2012 to 2023

**DOI:** 10.1093/inthealth/ihaf158

**Published:** 2026-01-07

**Authors:** Na Wang, AiLing Hu, Zexin Wang, Xiaojie Yu, Ying Wei, PingPing Song

**Affiliations:** Department of Pharmacy, The First Hospital of Qinhuangdao, Qinhuangdao 066000, China; Department of Pharmacy, The First Hospital of Qinhuangdao, Qinhuangdao 066000, China; Department of Pharmacy, The First Hospital of Qinhuangdao, Qinhuangdao 066000, China; Department of Pharmacy, The First Hospital of Qinhuangdao, Qinhuangdao 066000, China; Department of Pharmacy, The First Hospital of Qinhuangdao, Qinhuangdao 066000, China; Department of Pharmacy, The First Hospital of Qinhuangdao, Qinhuangdao 066000, China

**Keywords:** antimicrobial resistance, bloodstream infections, carbapenem resistance, *Klebsiella pneumoniae*, machine learning, whole-genome sequencing

## Abstract

**Background:**

Bloodstream infections (BSIs) caused by *Klebsiella pneumoniae* pose a significant global health burden, complicated by rising antimicrobial resistance (AMR). This study aimed to characterize resistance patterns, identify predictors of carbapenem resistance, and develop a machine learning model to predict patient outcomes.

**Methods:**

In a retrospective analysis of 109 279 *K. pneumoniae* BSIs from tertiary hospitals in China (2012–2023), 11 000 isolates underwent whole-genome sequencing (WGS) and antimicrobial susceptibility testing. Cox proportional hazards and logistic regression models identified predictors of 30-day mortality and carbapenem-resistant *K. pneumoniae* (CRKP), respectively. A random forest model predicted AMR trends and outcomes, evaluated by accuracy, precision, recall, and ROC-AUC using R Studio (R Studio, Inc., Boston, MA, USA).

**Results:**

Carbapenem resistance occurred in 32.3% of isolates, with rates of 41.9% for third-generation cephalosporins and 41.2% for fluoroquinolones. Among sequenced isolates, ST11 with *bla*KPC was the dominant CRKP genotype (12.0%). *bla*KPC (OR 3.97, 95% CI 3.10–5.11) and *bla*NDM (OR 2.80, 95% CI 2.07–3.71) strongly predicted carbapenem resistance; ICU admission predicted 30-day mortality (HR 2.10, 95% CI 1.80–2.46, p<0.001). Mortality was higher in CRKP (40.2%) vs. susceptible cases (21.5%). The random forest model achieved 89.2% accuracy and 0.92 ROC-AUC, with drug share, age, and CRKP status as top predictors.

**Conclusions:**

CRKP, especially ST11-*bla*KPC, drives excess mortality. Key predictors highlight the urgency for enhanced AMR surveillance and targeted therapy.

## Introduction

*Klebsiella pneumoniae* is a leading cause of bloodstream infections (BSIs) globally, significantly impacting hospital settings with high morbidity and mortality.^1^ With the increasing antimicrobial resistance (AMR) to carbapenems, cephalosporins and aminoglycosides, treatment options following the blood infection have been greatly limited.^2^ Song et al.^1^ revealed that 800 000 people died from *K. pneumoniae* infections, where 80% were linked to AMR, much of which occurred in South Asia and sub-Saharan Africa. Antimicrobial-resistant *K. pneumoniae* strains have become highly persistent, with the convergence of virulence and resistance factors leading to profound public health implications.^3^ The epidemiologic and predictive modeling strategies regarding the rise of carbapenem-resistant *K. pneumoniae* (CRKP) BSIs in China over the past decade are still necessary.^4^ Clinical risk factors such as prior antibiotic exposure, comorbidities and hospital-acquired origin further exacerbate resistance and mortality risks, with inappropriate initial therapy shown to raise mortality from 8.8% to 77.2%.^5–7^

*Klebsiella pneumoniae* has substantial genomic diversity, with high-risk sequence type (ST) (11, 25, 101 and 307) being commonly found in infection clusters.^2,3^ Extended-spectrum beta-lactamase (ESBL) genes like *bla*CTX-M-15, and carbapenemase genes like *bla*KPC and *bla*NDM-1, have been associated with hospital outbreaks and high mortality rates.^8^ Carbapenemase genes such as *bla*KPC and *bla*NDM are strongly linked to excess mortality, with affected patients facing more than a threefold increased risk of death.^9,8^ Whole-genome sequencing (WGS) confirms marked genetic diversity in *K. pneumoniae* isolates, complicating outbreak tracking and resistance prediction,^10^ while recent Asian reports show >50% carbapenem resistance and 66% multidrug resistance.^11^ Additional plasmid-encoded virulence genes in bloodstream isolates are often distinct from those found in colonizing strains, also supporting the role of antimicrobial-resistant determinants in *K. pneumoniae* BSI pathogenicity.^12^ Thirty-day mortality rates were significantly higher in patients with CRKP BSIs, 43.7%, as compared with carbapenem-susceptible *K. pneumoniae* (CSKP).^13^ Significantly, premature infants with CRKP BSIs had associated persistent tachycardia (71.9%), fever (63.5%) and apnea (61.4%), and the highest mortality risks occurred with meningitis and necrotizing enterocolitis.^14^ Treatment of ceftazidime–avibactam (CAZ/AVI) improved survival and predicted 92% sensitivity and 85.7% specificity for mortality. These findings underscore the need for robust predictive models to identify high-risk patients and inform antibiotic stewardship strategies.

Machine learning models have demonstrated potential for integrating genomic and clinical data to predict resistance in real time, although most prior work has been retrospective and limited in outcome assessment.^15–17^ The random forest (RF) algorithm, as a machine learning technique, offers promising features in predicting AMR patterns and patient outcomes in *K. pneumoniae* BSIs.^15,18–21^ A meta-analysis indicated that combination therapy notably improved survival in patients with CRKP BSIs if resistance prevalence was >50%.^22^ However, the treatment response remains uncertain and is strongly influenced by genetic and clinical factors, which require more sophisticated data-driven models. Traditional statistical methods^4^ like logistic regression have been used to predict CRKP BSI prognosis, but they tend to ignore combinations of the risk factors and never account for the interactions between the risk factors. With large-scale clinical, microbiological and genomic datasets integrated into RF models, predictive accuracy is improved by means of building reliable, real-time risk stratification and early intervention.^16^ Considering the growing AMR burden in China, effective strategies in patient management and hospital infection control policy are in high demand, and a longitudinal machine learning approach has been implemented to realize this goal.

The current study aims to (i) characterize AMR patterns in *K. pneumoniae* BSIs, (ii) identify genomic and clinical predictors of carbapenem resistance and 30-d mortality and (iii) develop a machine learning framework for predicting patient outcomes. It is hypothesized that carbapenem resistance is primarily driven by genomic determinants such as *bla*KPC and *bla*NDM, while clinical severity factors, including ICU admission, strongly predict 30-d mortality.

## Methodology

### Study design

A retrospective longitudinal study was conducted, utilizing clinical and genomic data from tertiary hospitals in China from 2012 to 2023. We performed this analysis by integrating electronic medical records (EMRs), antimicrobial susceptibility testing (AST) data and WGS data, assessing AMR patterns of *K. pneumoniae* BSIs. The resistance predictions, as well as the patient outcomes, were predicted using a RF algorithm. Data were collected from 18 tertiary hospitals across Beijing, Shanghai, Guangzhou, Wuhan and Chengdu. Hospitals differed in case mix, with the Beijing and Shanghai centers managing higher proportions of immunocompromised and ICU patients, while the Wuhan and Chengdu sites reported higher rates of community-onset bacteremia.

### Study population

To assess whether the associations between predictors of resistance and patient outcomes could be estimated with 90% power at a 95% confidence level, a power analysis using G*Power (Heinrich Heine University Düsseldorf, Düsseldorf, Germany) was run with 109 279 cases. A total of 109 279 patients with *K. pneumoniae* BSIs from multiple tertiary hospitals in China were collected with data.

***Inclusion criteria***

- Patients aged ≥18 y at admission.
- *Klebsiella pneumoniae* BSIs confirmed by at least one positive blood culture using the BACTEC automated system.
- Species identification via MALDI-TOF MS or PCR.
- AST results for carbapenems, cephalosporins, aminoglycosides, fluoroquinolones and β-lactam/β-lactamase inhibitors.
- Availability of clinical metadata, including demographics, comorbidities, treatment history and patient outcomes.
- WGS data for at least 10% of isolates.
- Hospitalization for at least 48 h to assess treatment response.

***Exclusion criteria***

- Patients aged <18 y or with incomplete demographic data.
- Polymicrobial infections where *K. pneumoniae* was not the primary pathogen. Polymicrobial exclusion criteria applied specifically to BSIs where *K. pneumoniae* was not the dominant isolate. Primary pathogen designation was based on quantitative culture results, clinical correlation with infection site and concordant AST.
- Cases missing AST results for at least three major antibiotic classes.
- Blood culture contaminants not confirmed as *K. pneumoniae* by molecular methods.
- Duplicate records within the same hospital stay.
- Patients in palliative care without active antimicrobial treatment.

### Description of data

Data were extracted from EMRs, microbiology laboratory databases and genomic sequencing repositories. The dataset contained 40 variables, including:

- **Demographics:** age, gender, birthdate.
- **Clinical parameters:** admission and discharge data, hospital stay duration, diagnosis, surgery records and medication data.
- **Financial data:** treatment costs, drug expenses and antibiotic cost percentage.

### Ethical considerations

This study was approved by the Peking Union Medical College Hospital Institutional Review Board (IRB No. 2021-CP-045), with waiver of informed consent due to retrospective design. Data were anonymized and stored following Chinese medical data privacy regulations.

### Sampling and isolation of *Klebsiella pneumoniae*

From 109 279 cases, 11 000 non-duplicate isolates were randomly selected for WGS, stratified by hospital, year and resistance phenotype to ensure balanced representation across sites and temporal trends. Blood cultures were processed using the BACTEC FX40 Blood Culture System (Becton, Dickinson and Company, USA). Positive cultures were subcultured on MacConkey agar and blood agar and incubated at 37°C for 18–24 h. Colonies suggestive of *K. pneumoniae* were identified using MALDI-TOF MS (Bruker Daltonics, Germany) or PCR. AST was conducted using the VITEK 2 Compact System (bioMérieux, France) following Clinical and Laboratory Standards Institute guidelines. WGS was performed on an Illumina NovaSeq 6000 platform (Illumina, USA) and AMR gene identification was conducted using ResFinder (Center for Genomic Epidemiology, Technical University of Denmark, Lyngby, Denmark).

### Genomic determinants analysis

WGS underwent quality control using FastQC (Babraham Bioinformatics, Babraham Institute, Cambridge, UK) and adapter removal with Trimmomatic (Usadel Lab, Aachen, Germany). Reads were assembled via SPAdes (v3.15) (Center for Algorithmic Biotechnology, St. Petersburg State University, St. Petersburg, Russia) and annotated using Prokka (v1.14.6) (Victorian Bioinformatics Consortium, Melbourne, VIC, Australia). Multilocus sequence typing (MLST v2.19 (Center for Genomic Epidemiology, Technical University of Denmark, Lyngby, Denmark)) and plasmid profiling (PlasmidFinder v2.1 (Center for Genomic Epidemiology, Technical University of Denmark, Lyngby, Denmark)) were performed, while antimicrobial-resistant and virulence genes were identified using ResFinder (v4.1) and VFDB (Virulence Factor Database) (Institute of Pathogen Biology, Chinese Academy of Medical Sciences, Beijing, China), respectively. Phylogenetic analysis involved Roary (v3.13) (Wellcome Sanger Institute, Hinxton, Cambridge, UK) for core genome alignment and IQ-TREE (v2.0) (University of Vienna, Vienna, Austria) for maximum-likelihood tree construction. DESeq2 (European Molecular Biology Laboratory (EMBL), Heidelberg, Germany) was used for batch effect correction and normalization. Comparative genomic analysis examined the distribution of antimicrobial-resistant genes, plasmid-mediated resistance and virulence factors across various clinical settings and infection types.

### Random forest framework

A RF model integrated clinical, microbiological and genomic features, with missing data imputed and key predictors selected via recursive feature elimination. The RF model was developed de novo for this study using Python scikit-learn (v1.2.2, scikit-learn developers, Paris, France), and hyperparameters were tuned via grid search; no previously published models were reused. The dataset was stratified into 70% training and 30% validation sets, with standardized continuous variables and one-hot encoded categorical data, and outliers removed using isolation forest and z-score methods.

**RF Algorithm**

**Input:** Dataset $\{ ( {{x}_i,{y}_i} )\} _{i = 1}^N $, T, m

**Output**: Trained Model, ${f}_{\mathrm{\theta }} $

**Step 1:** Initialize model with T, max. depth, min. samples split

**Step 2**: For each tree t = 1 to T

Apply bootstrap sampling, feature selection

**Step 3**: Train Decision Tree


\begin{eqnarray*}
{\mathrm{Gini}} = 1 - \mathop \sum \nolimits_{{\mathrm{i}} = 1}^{\mathrm{C}} {\mathrm{p}}_{\mathrm{i}}^2;\quad {\mathrm{Entropy}} &=& - \mathop \sum \nolimits_{{\mathrm{i}} = 1}^{\mathrm{C}} {{\mathrm{p}}}_{\mathrm{i}}{\log }_2\left( {{{\mathrm{p}}}_{\mathrm{i}}} \right);\nonumber\\
&&{\mathrm{\hat{y}}} = \frac{1}{{\mathrm{T}}}\mathop \sum \nolimits_{{\mathrm{t}} = 1}^{\mathrm{T}} {{\mathrm{f}}}_{\mathrm{t}}\left( {\mathrm{x}} \right)
\end{eqnarray*}

**Step 4**: Apply Hyperparameter Tuning

**Step 5**: Evaluate Model and Classification Metrics


\begin{eqnarray*}
{\mathrm{AUC}} &=& \mathop \int \nolimits_{ - \infty }^\infty {\mathrm{P}} \left( {{\mathrm{TPR}} > {\mathrm{FPR}}} \right) {\rm d}( {{\mathrm{FPR}}});\nonumber\\
{\phi }_{\mathrm{i}} &=& \mathop \sum \nolimits_{{\mathrm{S}} \subseteq {\mathrm{N}} \setminus \left\{ {\mathrm{i}} \right\}} \frac{{\left| {\mathrm{S}} \right|!\left( {\left| {\mathrm{N}} \right| - \left| {\mathrm{S}} \right| - 1} \right)!}} {{\left| {\mathrm{N}} \right|!}} \left[ {{\mathrm{f}} \left( {{\mathrm{S}} \cup \left\{ {\mathrm{i}} \right\}} \right) - {\mathrm{f}} \left( {\mathrm{S}} \right)} \right]
\end{eqnarray*}

**Step 6**: Return ${f}_{\mathrm{\theta }}$

### Statistical analysis

Statistical analyses were conducted using R (v4.2.2, R Foundation for Statistical Computing, Vienna, Austria) and Python (v3.9, Python Software Foundation, Wilmington, DE, USA). Descriptive statistics summarized patient characteristics and AMR trends, reporting means with SDs or medians with IQRs for continuous variables and frequencies with percentages for categorical data. Kaplan–Meier survival analysis with the log-rank test compared mortality rates, while Cox proportional hazard models estimated HRs with 95% CIs. Multivariate logistic regression adjusted for confounders, with variables selected based on clinical relevance and univariate significance (p<0.05). Model assumptions were assessed using Hosmer–Lemeshow tests, and collinearity was checked via variance inflation factors. All tests were two-tailed, with p<0.05 considered statistically significant. Data visualization, including survival curves and correlation plots, was generated using ggplot2 (R) (R Foundation for Statistical Computing, Vienna, Austria) and Matplotlib (Matplotlib Development Team, USA).

## Results

The participants (N=109 279) had a mean age of 59.2 y, with 40.8% being aged ≥65 y. Hypertension and chronic kidney disease were more common in males, while females had slightly shorter hospital stays (median 9 vs 10 d) (Table S1). Notably, females had a slightly shorter hospital stay (median 9 vs 10 d) and were enrolled in clinical pathways.

WGS data were available for 10.1% of isolates, revealing significant differences in sequence type distribution and in key antimicrobial-resistant genes between hospital-acquired and community-acquired isolates (Table 1). Plasmid profiles (IncC) and specific virulence genes (rmpA/rmpA2) also exhibited higher prevalence in hospital-acquired strains. Genomic screening identified additional resistance determinants, including *bla*VIM, in 4.1% of sequenced isolates, frequently co-occurring with *bla*NDM, highlighting the polyresistant genetic background of hospital-acquired strains. Of sequenced isolates, 6.7% were hypermucoviscous, predominantly linked to rmpA/rmpA2-positive strains, and were associated with higher rates of liver abscess.

**Table 1.** Microbiological and genomic characteristics of *K. pneumoniae* isolates

Parameter	Overall (%)	Hospital-acquired (%)	Community-acquired (%)	Significance
WGS-confirmed isolates (subset, n=11 000)	10.1	6500 (59.1)	4500 (40.9)	—
MLST				
ST11	2350 (21.4)	1780 (27.4)	570 (12.7)	0.002*
ST15	1980 (18.0)	1100 (17.0)	880 (19.6)	0.145
ST307	1210 (11.0)	700 (10.8)	510 (11.3)	0.493
Other STs (<5% each)	5460 (49.6)	2920 (44.9)	2540 (56.4)	0.001*
PlasmidFinder (+) for plasmids				
IncF	3960 (36.0)	2310 (35.5)	1650 (36.7)	0.678
IncC	2420 (22.0)	1620 (24.9)	800 (17.8)	0.012*
Other plasmids (<5% each)	4620 (42.0)	2570 (39.6)	2050 (45.6)	0.025*
Virulence genes (VFDB)				
rmpA/rmpA2	1870 (17.0)	1200 (18.5)	670 (14.9)	0.041*
iuc/iro	2310 (21.0)	1380 (21.2)	930 (20.7)	0.781
Antimicrobial-resistant genes (ResFinder)				
*bla*KPC	3020 (27.5)	2190 (33.7)	830 (18.4)	0.001*
*bla*NDM	1650 (15.0)	1330 (20.5)	320 (7.1)	0.001*
*bla*CTX-M (ESBL)	4620 (42.0)	2970 (45.7)	1650 (36.7)	0.007*
Phylogenetic clusters (Roary + IQ-TREE)	6 major clusters			

ESBL: extended-spectrum beta-lactamas; MLST: multilocus sequence typing; ST: sequence type; WGS: whole-genome sequencing.

*p<0.05 is significant.

**Figure 1.**
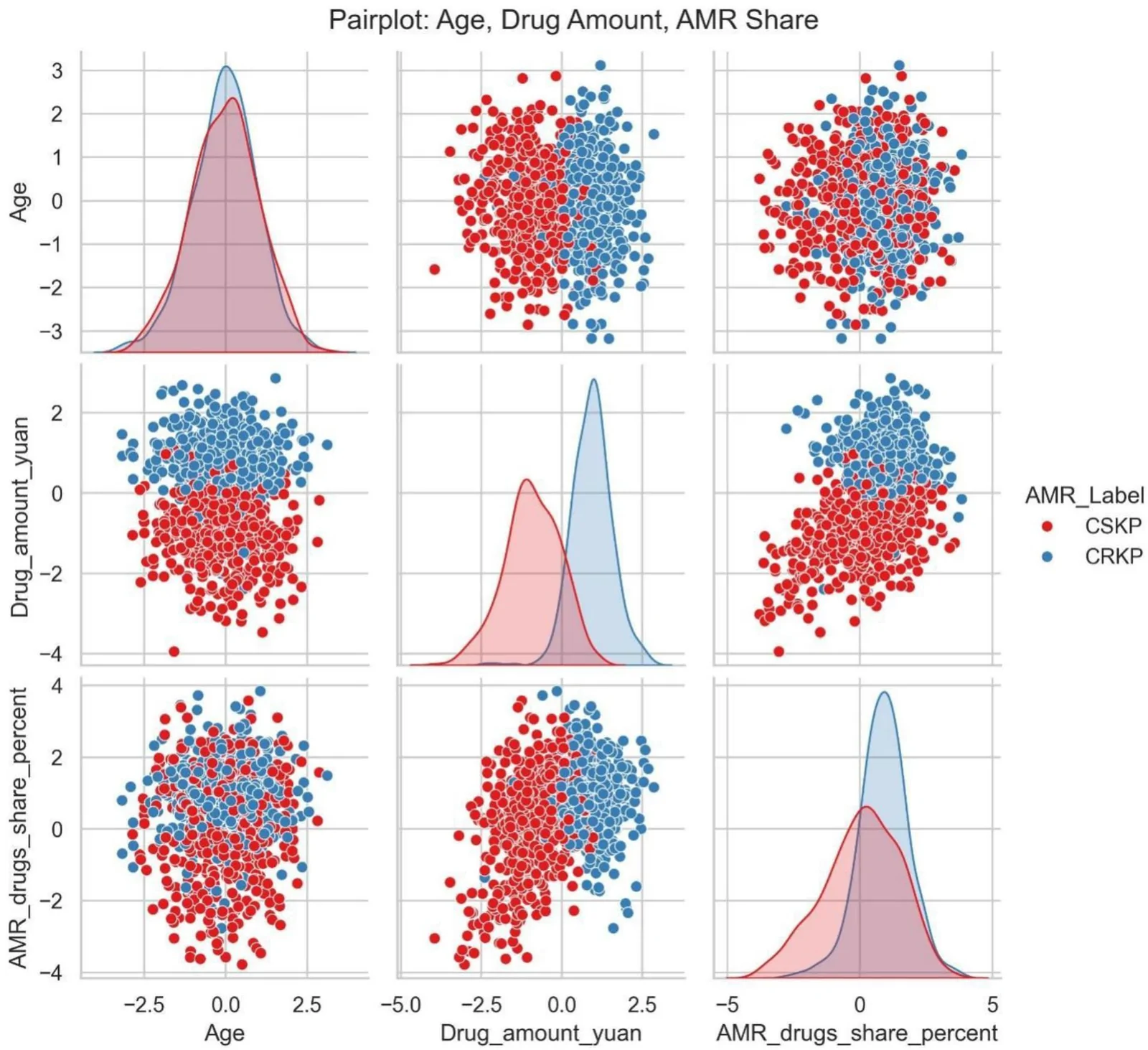
Pairplot visualizing the relationships between age, drug amount (yuan) and AMR drug share percentage, with density distributions for each variable and scatter plots colored by AMR label, distinguishing CRKP and CSKP. AMR: antimicrobial resistance; CRKP: carbapenem-resistant *Klebsiella pneumoniae*; CSKP: carbapenem-susceptible *Klebsiella pneumoniae.*

Carbapenem resistance was observed in 32.3% of *K. pneumoniae* isolates, with even higher resistance rates for third-generation cephalosporins (41.9%) and fluoroquinolones (41.2%) (Table S2). Resistance to fluoroquinolones (42%) was calculated across all isolates, including both CRKP and CSKP groups (Figure 1). Notably, colistin and tigecycline remained the most effective agents, with susceptibility rates of 85.1% and 79.6%, respectively. Among WGS-confirmed isolates (n=11 000), ST11 with *blaKPC* was the most prevalent genotype associated with carbapenem resistance (12.0%), while ST15 with *bla*CTX-M exhibited high ESBL positivity but remained carbapenem-susceptible (8.9%) (Table S4). Notably, a majority (68.9%) displayed mixed resistance phenotypes, reflecting substantial genetic diversity in antimicrobial-resistant determinants. Among carbapenem-resistant isolates, susceptibility rates were 72.4% for cefiderocol, 61.3% for eravacycline, 55.8% for minocycline and 68.9% for aztreonam–avibactam, confirming retained activity of several novel or repurposed agents.

The RF model achieved optimal performance with 200 estimators, a maximum depth of 20 and bootstrap enabled, yielding a crossvalidation area under the receiver operating characteristic curve (ROC-AUC) of 0.853±0.007.

**Figure 2.**
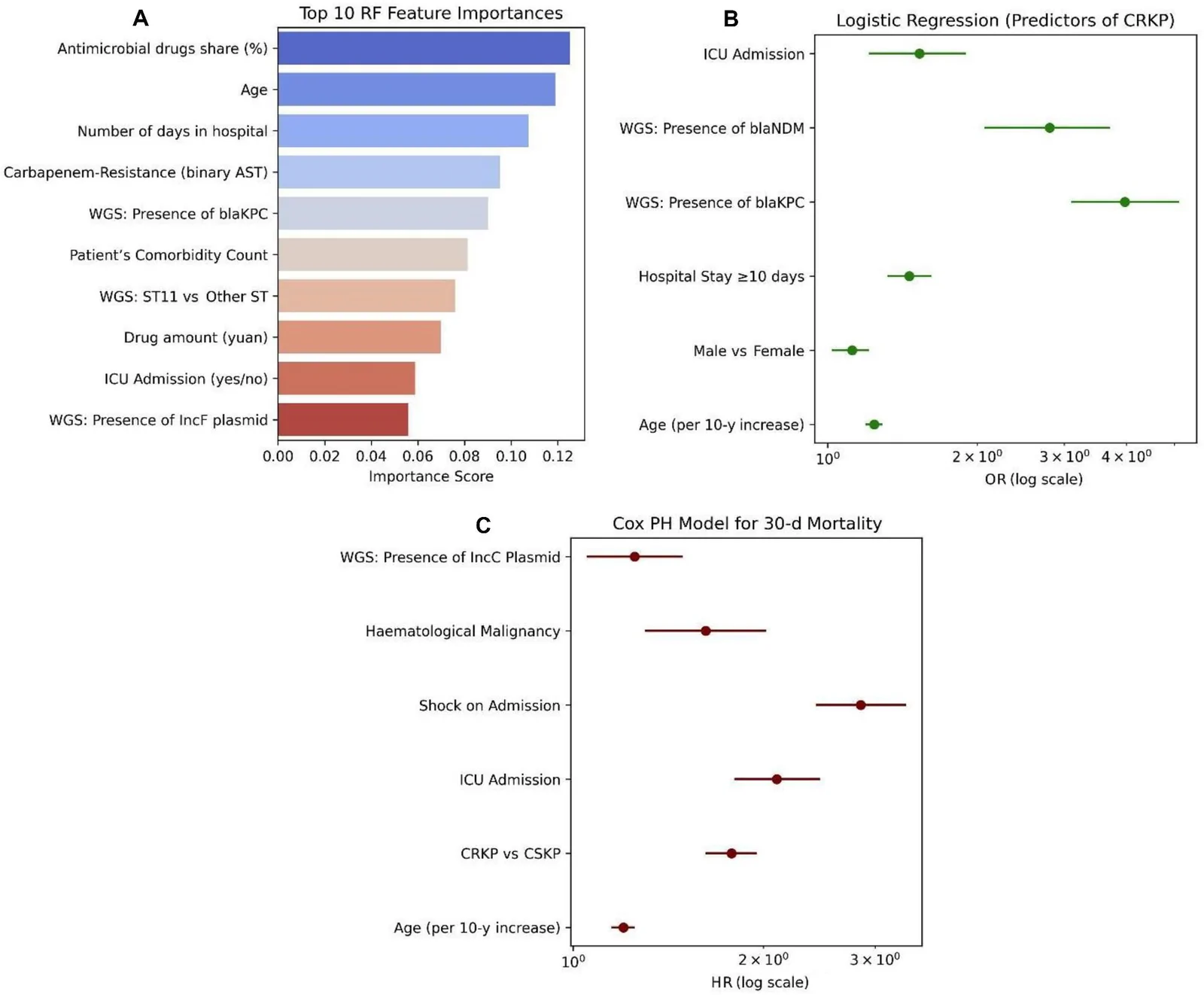
(A) Top 10 feature importances in the RF model for predicting antimicrobial resistance and clinical outcomes, (B) logistic regression model showing key predictors of CRKP with ORs on a log scale, and (C) Cox PH model identifying significant risk factors for 30-d mortality with HRs on a log scale. AST: antimicrobial susceptibility testing; CRKP: carbapenem-resistant *Klebsiella pneumoniae*; PH: proportional hazards; RF: random forest; ST: sequence type; WGS: whole-genome sequencing.

According to Figure 2, the top predictors were clearly defined as follows: antimicrobial drug share (%) represented the proportion of total inpatient drug costs attributable to antimicrobials; age indicated years at admission; number of days in hospital referred to total length of stay; carbapenem-resistance (binary AST) was coded as resistant to ≥1 carbapenem vs susceptible; WGS: presence of *bla*KPC denoted detection of the *bla*KPC gene; patient’s comorbidity count reflected the number of chronic conditions based on ICD-10 codes; WGS: ST11 vs other ST-classified isolates as ST11 or non-ST11; drug amount (yuan) captured cumulative inpatient antibiotic expenditure; ICU admission (yes/no) was coded as a binary variable; and WGS: presence of IncF plasmid indicated detection of the IncF plasmid. The RF model identified antimicrobial drug share (%) as the strongest predictor of resistance, followed by age and hospital stay duration (Figure 2A). Notably, genomic factors such as WGS-confirmed *bla*KPC presence and ST11 classification were also among the top predictors, emphasizing the role of both clinical and genetic variables in AMR patterns.

Logistic regression analysis revealed that the presence of *bla*KPC (OR 3.97, 95% CI 3.10 to 5.11) and *bla*NDM (OR 2.80, 95% CI 2.07 to 3.71) were the strongest predictors of CRKP (Table 2 and Figure 2B). Other significant risk factors included ICU admission (OR 1.53, p<0.001) and hospital stay ≥10 d (OR 1.46, p<0.001), highlighting the combined impact of genetic and clinical factors on resistance. Previous antibiotic exposure (within 90 d) was significantly associated with CRKP infection (OR 1.71, 95% CI 1.42 to 2.06), while prior hospitalization in the past year also increased risk (OR 1.56, 95% CI 1.28 to 1.91).

**Table 2.** Logistic regression analysis for predictors of carbapenem-resistant *K. pneumoniae*

Variable	OR (95% CI)	p
Age (per 10-y increase)	1.24 (1.19–1.29)	<0.001*
Male vs female	1.12 (1.02–1.21)	0.015*
Hospital stay ≥10 d	1.46 (1.32–1.62)	<0.001*
WGS: presence of *bla*KPC	3.97 (3.10–5.11)	<0.001*
WGS: presence of *bla*NDM	2.80 (2.07–3.71)	<0.001*
ICU admission	1.53 (1.21–1.90)	<0.001*
Constant	–	0.022*

WGS: whole-genome sequencing.

*p<0.05 is significant.

Cox proportional hazards analysis demonstrated that septic shock upon admission (HR 2.85, 95% CI 2.42 to 3.36) and ICU admission (HR 2.10, 95% CI 1.80 to 2.46) were the strongest predictors of 30-d mortality (Table 3 and Figure 2C). Notably, CRKP infection was associated with a 78% increased mortality risk compared with CSKP (HR 1.78, 95% CI 1.62 to 1.95, p<0.001), reinforcing the clinical impact of carbapenem resistance. Subgroup analysis after the introduction of CAZ/AVI (2015) and meropenem–vaborbactam (2018) showed modest declines in mortality among CRKP cases (45.8% pre-2015 vs 38.6% post-2018, p=0.041), although resistance prevalence remained high across all years (Table S3).

**Table 3.** Cox proportional hazards model for 30-d mortality

Variable	HR (95% CI)	p
Age (per 10-y increase)	1.20 (1.15–1.25)	<0.001*
CRKP vs CSKP	1.78 (1.62–1.95)	<0.001*
ICU admission	2.10 (1.80–2.46)	<0.001*
Shock on admission	2.85 (2.42–3.36)	<0.001*
Hematological malignancy	1.62 (1.30–2.02)	<0.001*
WGS: presence of IncC plasmid	1.25 (1.05–1.49)	0.016*
Constant	–	0.031*

CRKP: carbapenem-resistant *K. pneumoniae*; CSKP: carbapenem-susceptible *K. pneumoniae*; WGS: whole-genome sequencing.

*p<0.05 is significant.

**Figure 3.**
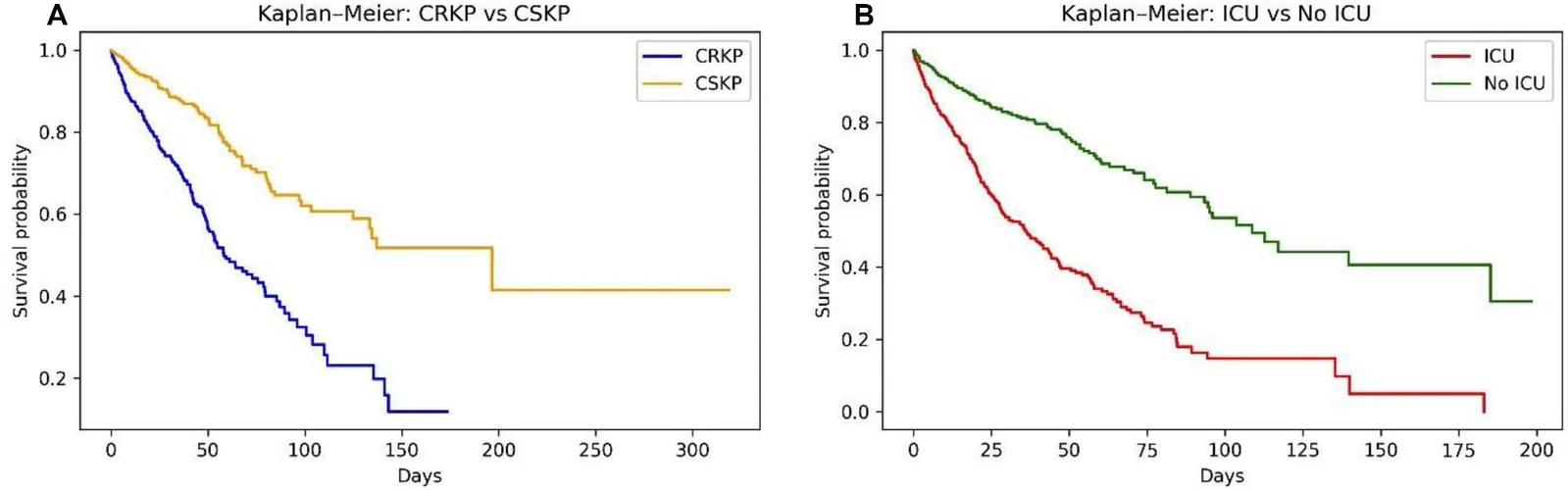
(A) Kaplan–Meier survival curves comparing CRKP and CSKP, showing significantly lower survival probability in CRKP cases, and (B) Kaplan–Meier survival curves comparing patients with and without ICU admission, indicating higher mortality risk among ICU-admitted patients. CRKP: carbapenem-resistant *Klebsiella pneumoniae*; CSKP: carbapenem-susceptible *Klebsiella pneumoniae.*

Kaplan–Meier survival analysis revealed significantly higher 30-d mortality rates in CRKP cases (40.2%) compared with CSKP (21.5%, p<0.001) (Table S5 and Figure 3A,B). ICU admission was associated with the highest mortality (48.3%), while older age (≥65 y) also significantly increased mortality risk (p=0.006). The final RF model achieved strong classification performance, with an accuracy of 89.2% and a ROC-AUC of 0.92, highlighting its predictive reliability (Table S6). Antimicrobial drug share (%), age and CRKP status were the top predictors of resistance and mortality, with SHAP analysis confirming the influence of ST11 and *bla*KPC presence in the classification of resistance.

## Discussion

The current study reinforces the significant burden of AMR in *K. pneumoniae* BSIs, with carbapenem resistance (32.3%) aligning with global trends. WGS revealed the dominance of ST11 carrying *bla*KPC, highlighting clonal persistence in resistance patterns. The 30-d mortality rate for CRKP cases (40.2%) underscores the severity of these infections and the need for improved therapeutic strategies. Machine learning models, notably the RF algorithm (ROC-AUC=0.92), effectively identified clinical and genomic predictors of resistance and mortality. Consistent with Long et al.,^23^ our findings confirm *K. pneumoniae* as a leading Gram-negative BSI pathogen, with high in-hospital mortality, particularly in patients with septic shock or requiring mechanical ventilation. Colistin-isolated susceptibility parallels those of polymyxin B, confirmed in similar rates of resistance (11.6%), thus proving to be a last-line treatment. Further supporting this relationship between drug- or antimicrobial-resistant *K. pneumoniae* and poor clinical outcomes, the overall mortality rate is 28.7%.^24–30^

Inappropriate initial antibiotic selection raises mortality dramatically from 8.8% to 77.2%, as shown by Ma et al.,^7^ emphasizing the importance of resistance in clinical outcomes. Similar to our findings, the 30-d mortality risk (HR 2.10) is significantly increased with ICU admission, where antimicrobial regimens in critically ill patients necessitate optimization. High carbapenem use has contributed to the increase of antimicrobial-resistant *K. pneumoniae*, making antimicrobial stewardship ever so important. Resistance patterns in *K. pneumoniae* are geographically specific, with Pezzani et al.^31^ reporting that carbapenem resistance in *K. pneumoniae* constitutes 62.8% of cases, while primarily being hospital-acquired in southern Europe. This finding is similar to our study’s results, where hospital-acquired isolates show a higher prevalence of *bla*KPC and *bla*NDM than community-acquired strains, indicating that region-specific AMR surveillance is necessary. As is the case with other staphylococci, ST11 has been associated with hospital epidemiologic dissemination, which supports global clonal expansion trends and results in persistent healthcare-associated transmission. Clinically, the RF model could be embedded into EMRs to provide real-time risk stratification at admission, guiding empiric therapy choices and early ICU triage. By integrating genomic predictors such as *bla*KPC/*bla*NDM with patient-level data, the model offers actionable outputs for antimicrobial stewardship programs and individualized treatment decisions.

According to Sajedi Moghaddam et al.,^32^  *K. pneumoniae* was found to represent 16% of Gram-negative BSIs, while 56% of isolates demonstrated imipenem resistance yet colistin susceptibility. The observed efficacy of colistin aligns with our research outcomes; however, the development of resistance to the antibiotic underscores the need to combine therapeutic approaches. High-risk units must implement targeted infection control measures because CRKP is prevalent in ICUs. Roach et al.^10^ found that intra-abdominal infections were the most common, while respiratory tract infections had the highest mortality. Our results also indicate elevated mortality in CRKP cases, especially among patients with septic shock (HR 2.85). The genetic heterogeneity in their study aligns with our findings, supporting the need for broader genomic surveillance to track evolving resistance mechanisms. Wang et al.^13^ identified prior quinolone exposure and inappropriate empirical therapy as key mortality risk factors, consistent with our logistic regression findings that prolonged hospital stays (≥10 d) and ICU admission increased CRKP risk. This highlights the importance of early and accurate pathogen identification to inform empirical treatment. The ongoing emergence of multidrug-resistant strains, as shown in our study, calls for integrated surveillance strategies that combine clinical and genomic data to refine treatment guidelines dynamically.

Waterlow et al.^17^ found higher AMR rates in older patients and regional prevalence variations, aligning with our findings that age ≥65 y was a significant mortality predictor (35.6% 30-d mortality). This highlights aging populations as an at-risk group requiring targeted interventions in high-prevalence areas. Wu et al.^33^ identified agranulocytosis lasting >20 d and septic shock as key mortality predictors, consistent with our results showing ICU admission and septic shock upon admission as significant risk factors. Yang et al.^34^ reported hypervirulent *K. pneumoniae* strains complicating treatment, even in the absence of *rmp* genes. This suggests alternative virulence regulation pathways that require further investigation to understand their clinical impact. In addition, recent evaluations of CAZ/AVI and meropenem–vaborbactam use demonstrate that timely access to novel agents reduces mortality among CRKP patients by up to 15%,^35,36^ underscoring the need to align predictive models with therapeutic decision support. Incorporating drug availability, formulary restrictions and cost data into machine learning frameworks may therefore bridge the gap between genomic risk prediction and real-world treatment outcomes.

### Practical implications

The findings of this study provide actionable opportunities for health systems and policymakers. Early identification of high-risk *K. pneumoniae* BSI patients through machine learning models enables optimized allocation of scarce ICU resources and tailored infection control strategies in high-prevalence wards. Hospital formulary planning can be guided by model outputs that forecast resistance trends, supporting more efficient procurement of last-line antibiotics. In addition, integration of genomic surveillance into predictive algorithms allows laboratories to rapidly flag clonal dissemination events such as ST11-*bla*KPC, thereby reducing the lag between detection and intervention. Embedding these models into antimicrobial stewardship programs also fosters data-driven decision support, ensuring empiric therapy is targeted without unnecessarily escalating broad-spectrum drug use.

### Limitations and future research

This study is limited by its retrospective design, which restricts causal inference, and by its restriction to tertiary hospitals in China, which may not capture patterns in community hospitals or other global regions. Genomic sequencing was performed on a subset of isolates, which may not fully represent the diversity of circulating strains. Variability in clinical record completeness across sites may also have introduced residual confounding. Future research should validate these findings in prospective, multicenter cohorts, expand geographic scope to include diverse healthcare settings and integrate real-time sequencing data to refine predictive accuracy. Incorporating additional antimicrobial classes and exploring novel algorithms such as gradient boosting or deep learning could further enhance clinical applicability and predictive performance.

### Conclusion

Our study enhances the understanding of *K. pneumoniae* BSIs by integrating large-scale clinical, microbiological and genomic data. Identifying key resistance predictors and mortality risk factors through statistical and machine learning models improves AMR surveillance and treatment precision. The high CRKP burden in hospital settings underscores the need for antimicrobial stewardship and infection control. Future research should refine predictive models using real-time genomic surveillance and explore novel therapeutic strategies to combat AMR.

## Supplementary Material

ihaf158_Supplemental_File

## Data Availability

The datasets generated and/or analysed during the current study are publicly available in the Dryad Digital Repository at: https://datadryad.org/share/IIGLDDBm1W5JIOkfPQmaXuz2YZKT-B9pMWhsyjxNuYs.
