# Supplementary material for: Antimicrobial resistance analysis of Klebsiella pneumoniae bloodstream infections based on a random forest algorithm: a longitudinal study based on data from tertiary hospitals in China from 2012 to 2023
Source: Int Health. 2026 Jan 7;18(5):773–81. doi: 10.1093/inthealth/ihaf158 (PMC13530284; doi:10.1093/inthealth/ihaf158)
Supplement: Supplementary file 1 [file ihaf158_supplemental_file.docx]

**Table S1.** Baseline Characteristics of the Study Population

| **Variable** | **Overall (N=109,279)** | **Male (n=58,774)** | **Female (n=50,505)** | **p-value** |
| --- | --- | --- | --- | --- |
| **Age, years** (mean ± SD) | 59.2 ± 15.7 | 60.1 ± 15.2 | 57.9 ± 16.3 | 0.001* |
| **Age ≥ 65 years**, n (%) | 44,582 (40.8) | 25,111 (42.7) | 19,471 (38.5) | 0.003* |
| **Hospital Stay, days (median [IQR])** | 10 [7–15] | 10 [7–15] | 9 [6–14] | 0.011* |
| **Comorbidities**, n (%) |  |  |  |  |
| – Diabetes | 17,801 (16.3) | 9,830 (16.7) | 7,971 (15.8) | 0.112 |
| – Hypertension | 25,490 (23.3) | 14,112 (24.0) | 11,378 (22.5) | 0.045* |
| – Chronic Kidney Disease | 7,409 (6.8) | 4,319 (7.3) | 3,090 (6.1) | 0.029* |
| – Malignancy (Solid Tumour) | 9,261 (8.5) | 4,803 (8.2) | 4,458 (8.8) | 0.184 |
| – Haematological Malignancy | 4,128 (3.8) | 2,180 (3.7) | 1,948 (3.9) | 0.321 |
| **Insurance Type**, n (%) |  |  |  |  |
| – Urban Employee Medical Insurance | 45,928 (42.0) | 23,745 (40.4) | 22,183 (43.9) | 0.047* |
| – Urban & Rural Residents | 36,012 (33.0) | 20,120 (34.2) | 15,892 (31.5) | 0.059 |
| – Out-of-Province | 15,882 (14.5) | 8,966 (15.2) | 6,916 (13.7) | 0.102 |
| – Self-Pay / Uninsured | 11,457 (10.5) | 5,943 (10.1) | 5,514 (10.9) | 0.221 |
| Clinical Pathway Enrolment, n (%) | 52,140 (47.7) | 27,109 (46.1) | 25,031 (49.6) | 0.037* |

Note: *mean ± standard deviation (SD), median [interquartile range], or frequencies (percentages). p-values < 0.05 are marked with an asterisk (*).*

**Table S2.** Antibiotic Susceptibility Profiles of *K. pneumoniae*

| **Antibiotic Class** | **Susceptible (%)** | **Intermediate (%)** | **Resistant (%)** |
| --- | --- | --- | --- |
| **Carbapenems** (e.g.,Meropenem) | 70,699 (64.7) | 3,278 (3.0) | 35,302 (32.3) |
| **Cephalosporins** (3rd-Gen) | 59,207 (54.2) | 4,321 (3.9) | 45,751 (41.9) |
| **Aminoglycosides** (e.g., Amikacin) | 64,393 (58.9) | 9,240 (8.5) | 35,646 (32.6) |
| **Fluoroquinolones** (e.g., Ciprofloxacin) | 58,914 (53.9) | 5,364 (4.9) | 44,999 (41.2) |
| **β-lactam/β-lactamase inhibitors** (e.g., Piperacillin-Tazobactam) | 79,578 (72.8) | 4,855 (4.4) | 24,846 (22.7) |
| **Colistin** | 92,893 (85.1) | 4,371 (4.0) | 12,015 (11.0) |
| **Tigecycline** | 87,009 (79.6) | 2,730 (2.5) | 19,540 (17.9) |

Note: *All data are total counts and percentages of the total 109,279 isolates tested.*

**Table S3.** Distribution of Genotype-Phenotype Combinations (Subset n=11,000 WGS-Confirmed)

| **Genotype (ST + Key AMR Genes)** | **Phenotypic Resistance (Carbapenem, Cephalosporin, etc.)** | **n (%)** |
| --- | --- | --- |
| ST11 + bla(KPC) | Carbapenem-Resistant, ESBL Negative | 1,320 (12.0) |
| ST11 + bla(KPC) + bla(CTX-M) | Carbapenem-Resistant, ESBL Positive | 460 (4.2) |
| ST15 + bla(CTX-M) | ESBL Positive, Carbapenem-Susceptible | 980 (8.9) |
| ST307 + bla(NDM) | Carbapenem-Resistant, ESBL Negative | 660 (6.0) |
| Other ST + mixed AMR genes | Mixed Phenotypes | 7,580 (68.9) |

**Table S4.** Kaplan-Meier Survival Estimates by Key Variables

| **Group** | **n** | **30-day Mortality (%)** | **Log-Rank p-value** |
| --- | --- | --- | --- |
| **CRKP vs. CSKP** |  |  | <0.001* |
| – CRKP | 47,500 | 40.2 |  |
| – CSKP | 61,779 | 21.5 |  |
| **ICU Admission** |  |  | <0.001* |
| – Yes | 29,012 | 48.3 |  |
| – No | 80,267 | 18.9 |  |
| **Age ≥ 65 years** |  |  | 0.006* |
| – Yes | 44,582 | 35.6 |  |
| – No | 64,697 | 24.1 |  |

**Table S5.** Summary of Survival Analysis and Random Forest Classification Metrics

| **Metric/Outcome** | **Value / Description** |
| --- | --- |
| Mean Follow-up (days) | 45.2 ± 12.6 |
| Overall 30-day Mortality | 28.7% |
| Random Forest Accuracy | 89.2% |
| Random Forest Precision | 86.4% |
| Random Forest Recall | 83.9% |
| Random Forest F1-Score | 85.1% |
| Random Forest ROC-AUC | 0.92 |
| Key Predictors (RF Feature Ranking) | (1) AMR Share, (2) Age, (3) CRKP |
| Interpretability (SHAP) | Positive “Top Features”: AMR share, ST11, bla<sub>KPC</sub> presence |

Note: *Overall metrics reflect final model performance on the 30% validation set. The survival data correspond to patients who had complete 30-day outcome information (n ~ 105,000 after exclusions).*
